# *In Vitro* Osteogenic Differentiation of Human Mesenchymal Stem Cells from Jawbone Compared with Dental Tissue

**DOI:** 10.1007/s13770-017-0071-0

**Published:** 2017-08-28

**Authors:** Linda F. Pettersson, Paul J. Kingham, Mikael Wiberg, Peyman Kelk

**Affiliations:** 1 Department of Integrative Medical Biology, Section for Anatomy, Umeå University, 90187 Umeå, Sweden; 2 Department of Surgical and Perioperative Sciences, Section for Hand and Plastic Surgery, Umeå University, 90185 Umeå, Sweden; 3 Department of Odontology, Section for Oral and Maxillofacial Surgery, Umeå University, 90187 Umeå, Sweden

**Keywords:** Stem cells from apical papilla, Dental pulp stem cells, Periodontal ligament stem cells, Stem cells from jawbone, Osteogenic differentiation

## Abstract

Autologous bone transplantation is the current gold standard for reconstruction of jawbone defects. Bone regeneration using mesenchymal stem cells (MSC) is an interesting alternative to improve the current techniques, which necessitate a second site of surgery resulting in donor site morbidity. In this study, we compared the osteogenic ability of jawbone MSC (JB-MSC) with MSC from tissues with neural crest origin, namely, the dental pulp, apical papilla and periodontal ligament. All four types of MSC were isolated from the same patient (n = 3 donors) to exclude inter-individual variations. The MSC growth and differentiation properties were characterized. The osteogenic differentiation potential in each group of cells was assessed quantitatively to determine if there were any differences between the cell types. All cells expressed the MSC-associated surface markers CD73, CD90, CD105, and CD146 and were negative for CD11b, CD19, CD34, CD45 and HLA-DR. All cell types proliferated at similar rates, exhibited similar clonogenic activity and could differentiate into adipocytes and osteoblasts. An alkaline phosphatase assay, OsteoImage™ assay for mineralization and qRT-PCR measuring the genes *runx2*, *ALP* and *OCN*, indicated that there were no significant differences in the osteogenic differentiation ability between the various MSCs. In conclusion, we show that from a small segment of jawbone it is possible to isolate sufficient quantities of MSC and that these cells can easily be expanded and differentiated into osteoblasts. JB-MSC appear to be good candidates for future bone regeneration applications in the craniofacial region.

## Introduction

Defects in the jawbone due to lack of bone (besides physiological resorption) can have multiple causes such as congenital defects, trauma, or pathologic conditions. Lack of bone can lead to functional problems with impaired speech, chewing and swallowing ability as well as esthetic changes [1, 2]. This condition is often both disfiguring and disabling and has a major negative impact on the patients’ quality of life. Consequently, the need for reconstruction is high [3, 4]. Today the gold standard for functional reconstruction of mandible and maxilla is the use of autologous bone transplants. For small defects, free non-vascularized cortical bone transplants can be harvested from intraoral donor sites. If there is a need for a larger amount of bone the most common donor site is the iliac crest or fibula [5, 6]. The autologous bone grafts provide all the necessary factors for bone regeneration such as a scaffold material for osteoconduction, growth factors for osteoinduction and osteogenic cells. The autologous grafting procedure is however dependent on the possibility to harvest donor tissue and associated with a high risk of complications and donor site morbidity [7, 8].

Recent advances in the field of tissue engineering indicate that mesenchymal stem cells/multipotent stromal cells (MSC) could be an alternative to the conventional procedures and are associated with less morbidity, lower complication rates and improved function in bone regenerative treatments [9, 10]. The MSC can be isolated from various types of connective tissue and they show self-renewal and clonogenic activity and they have a capacity to differentiate into bone, cartilage and adipose lineages [11, 12]. MSC also have an ability to stimulate hematopoiesis and suppress immune reactions [13]. MSC have been identified to play a crucial role in various organs, preserving tissue homeostasis and regenerating damaged tissues [14]. Interestingly MSC exhibit different characteristics dependent on their harvest site [15, 16]. MSC isolated from bone marrow (BM-MSC) are the most widely studied, both in bone regeneration models and regarding their immunomodulatory effects. Thus BM-MSC have been tested therapeutically in various clinical trials and used to treat graft-versus-host disease following bone marrow transplantation [17, 18].

Isolation of MSC from jawbone (JB-MSC) was first described by Matsubara et al. [19]. The JB-MSC appear to have an osteogenic capacity comparable with BM-MSC from iliac crest but not as efficient chondrogenic and adipogenic capacity [16, 19]. The origin of the oro-facial bones as well as the teeth and the periodontal ligaments differs from the origin of the cranial vault and the rest of the axial and appendicular bones. The oro-facial bones as well as the teeth including periodontal tissue originates from neural crest cells whilst the cranial vault has a dual neural crest and mesodermal origin and the other axial and appendicular bones have an exclusively mesodermal origin [20]. Reconstruction of oro-facial defects using grafts from an oro-facial donor site results in a better outcome than when using a graft from another site [16]. Furthermore, several bone abnormalities are only observed in the oro-facial region suggesting that there might be a site-specific difference of progenitor cells in the bone marrow [16].

MSC isolated from dental tissue, with the same origin as jaw bone, include periodontal ligaments [21], dental pulp [22], exfoliated deciduous teeth [23], dental follicle [24], apical papilla [25, 26] and gingival tissue [27] have proven useful for a wide range of clinical applications [28, 29]. The dental MSC have much in common, such as a fast population doubling time and the ability to differentiate into multiple cell lineages. They also possess low immunogenicity and marked immunosuppressive activity. Nevertheless, there are also some differences between the various dental MSC types. For example stem cells of the apical papilla (SCAP) are derived from developing tissue and seem to represent a population of early stem cells, different from those found in mature tissues. SCAP show a higher proliferation rate and mineralization potential than dental pulp stem cells (DPSC) [21, 22, 26, 29, 30].

In this study, we isolated MSC from jawbone and compared these with 3 different types of MSC with dental origin; the SCAP, DPSC, and periodontal ligament stem cells (PDLSC). All cell types were compared from matched human donors. We subsequently characterized the growth properties of these cells and investigated their differentiation potential. By isolating all four cell types from the same donor we could ensure that the results were not affected by inter-donor variation in MSC properties. The overall aim was to determine if JB-MSC matched the dental MSC potential for use in bone regenerative therapies in the oro-facial region. If so, JB-MSC might be used as the optimal cell type for these applications, given their lower morbidity in harvesting.

## Materials and methods

### Ethical considerations

Written informed consent was obtained from patients. Collection, culture, storage and usage of all clinical isolates were approved by the local ethics committee for research at Umeå University (Dnr 2013-276-31M).

### Isolation of MSC

**Fig. 1:**
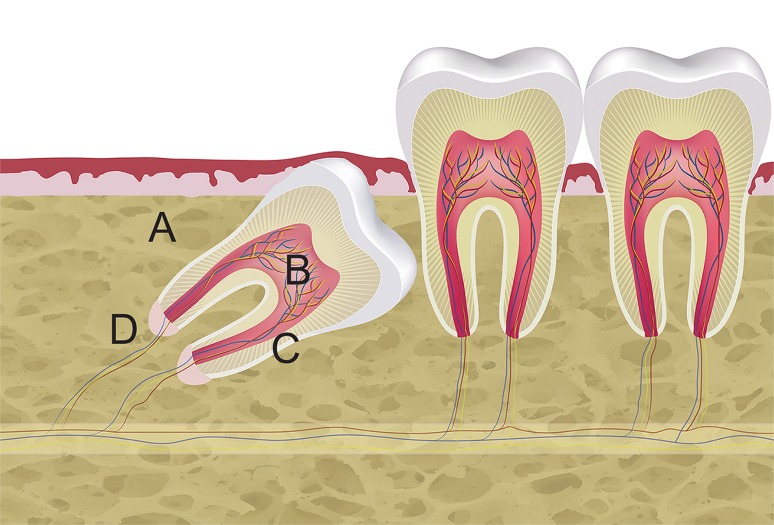
Harvest sites for MSCs from the oral cavity. Schematic illustration showing harvest sites of the various MSC: **A** Jawbone (JB-MSC), **B** dental pulp (DPSC), **C** periodontal ligament (PDLSC), **D** apical papilla (SCAP). The illustration was created in Adobe Illustrator CC (Adobe Systems Inc. San Jose, CA, USA)

SCAP, DPSC, PDLSC and JB-MSC were isolated from the same human donor. A schematic illustration of the location of these various MSC is shown in Fig. 1. Human impacted teeth (n = 3; two lower jaw third molars and one upper jaw canine) were surgically removed from 3 healthy patients (1 male and 2 females with mean age 17 years and range 11–20 years,) due to retention and lack of space. The surgical removal was conducted under local anesthesia at the Department of Oral- and Maxillofacial Surgery, Umeå University Hospital. After elevation of a full-thickness flap, the maxillary/mandibular bone covering the tooth crown from a buccal aspect was removed using a round bur under irrigation with sterile saline solution to prevent damage to the tissue. The bone, approximately 3 mm^3^ of dense cortical bone from each donor, (Fig. 1A) was immediately placed in a tube with Minimum Essential Medium alpha modification (α-MEM) and GlutaMax (GIBCO/Invitrogen, Carlsbad, CA) supplemented with 1% Antibiotic–Antimycotic solution (Sigma-Aldrich, St Louis, MO) and transferred to the laboratory within 4 h. After removing the tooth from the socket the tooth was placed in the same tube as the isolated cortical bone. Below a detailed description of the location and types of isolated MSCs are provided.

#### Jawbone MSC (JB-MSC)

A small piece, 3 mm^3^, of cortical bone covering the buccal aspect of the impacted tooth (Fig. 1A) in the mandible/maxilla was removed using a bur under irrigation with sterile saline solution. The bone was then particulated using a sharp scalpel and thereafter digested in a solution of 3 mg/ml Collagenase type I (Worthington Biochemicals Corp., Freehold, NJ) and 4 mg/ml Dispase II (Roche Diagnostic/Boehringer Mannheim Corp., Indianapolis, IN) in a 37 °C water bath for 60 min. Every 10 min the tubes were vortexed to help dissolve the tissue.

#### Dental pulp MSC (DPSC)

The dental pulp is the soft tissue inside a tooth. After separation of tooth crown from the root with a bur, the pulp tissue, approximately 5 mm^3^, was carefully removed using small files (Fig. 1B). The pulp tissue was then treated the same way as with the jawbone.

#### Periodontal ligament MSC (PDLSC)

The periodontal ligament is a group of specialized connective tissue fibers that essentially attach the tooth to the alveolar bone. It outlines the root surface of the tooth. The PDLSC were collected by scraping the tissue from the root surface (Fig. 1C) with a scalpel. The tissue, approximately 3 mm^3^, was then treated according to the same protocol (mincing and enzymatic digestions as described above).

#### Apical papilla MSC (SCAP)

The apical papilla is an approximately 5 mm^3^ soft tissue formation located at the apices of developing permanent teeth (Fig. 1D). To collect SCAP, the apical papilla was gently removed from the teeth with a scalpel, minced into small pieces and treated the same way as the other cell types.

After dissolving all tissues in the solution of Dispase II and Collagenase I for 60 min the solution was filtered using a 70 µm filter (Falcon, BD Labware, Franklin Lakes, NJ). The flow through was diluted with 4 ml of α-MEM + GlutaMAX supplemented with 15% Fetal Bovine Serum (FBS; GIBCO) and 1% Antibiotic–Antimycotic solution (SIGMA). The filter was further rinsed with 5 ml of Hanks Balanced Salt Solution (HBSS) to collect any remaining cells.

The solution was centrifuged for 7 min at 800*g* to obtain a small pellet of cells which was then resolved in 1 ml medium to a single cell suspension and diluted with a further 4 ml medium and transferred to a 25 cm^2^ tissue culture plastic flask (Thermo scientific, DK) and incubated at 37 °C with 5% CO_2_. The medium was changed after one day to remove the non–adherent cells. The medium was then left for 7 days and then changed continuously every other day until 95% confluency was achieved. Next, the cells were detached and transferred to 75 cm^2^ flasks (Thermo Scientific, DK) and allowed to grow until 90% confluency with changing of medium every other day. Upon confluence, the cells were detached using trypsin/EDTA solution (GIBCO) and were cryopreserved in a freezing solution of 10% Dimethyl sulfoxide (DMSO) (Sigma-Aldrich St Louis MO) with 90% FBS and stored at −80 °C. Cells between passages 1 and 3 were used in this study and all comparisons were made on cells at matching passage numbers.

### Characterization of MSC by flow-cytometry

The MSC-associated surface antigens of SCAP, DPSC, PDLSC and JB-MSC were characterized by flow cytometric analyses. All cells were analyzed at passage 1 and tested for positive MSC-associated surface markers (CD73, CD90, CD105, and CD146) and negative markers (CD11b, CD19, CD34, CD 45 and HLA-DR), to define the cells as MSC [29] according to the manufacturer’s protocol (BD Bioscience). All antibodies used for FACS analysis were PE-conjugated. Optimal concentrations of antibodies were calculated (1:25 for CD73, 1:33 for CD90, 1:25 for CD105, 1:25 for CD146, and 1:25 for the negative markers) and 50,000 cells for each analysis were chosen. As negative control, a corresponding isotype control was used for each sample (mouse IgG1, κ). Data were acquired using FACScalibur (BD Bioscience).

### Proliferation and colony forming unit-fibroblast assay

The cells were seeded in 25 cm^2^ culture flasks at a density of 2000 cells/cm^2^ and trypsinised, counted and replated every week (up to 5 weeks) in α-MEM containing 15% FCS (v/v) and 1% Penicillin–Streptomycin. From the weekly cell counts, cumulative population doublings (PD) was calculated according to the following formula: n = 3.32 (log [cell number after 7 days] − log [initial cell number]) + X, where n = the total PD number at end of each given subculture and X = the doubling level of the cells used to initiate the weekly subculture being quantitated.

Colony forming unit-fibroblast (CFU-F) assay was performed by establishing triplicates of each cell sample for all three donors in 12-well cell culture plates. Cells were seeded at a density of 50 cells per well. The cells were incubated for 14 days without change of medium. After 14 days the medium was removed and the cells were washed with phosphate-buffered saline (PBS; Invitrogen Life Technologies, Fisher Scientific) and then fixed for 20 min in 1% phosphate-buffered formalin made from paraformaldehyde (PFA). After the fixation step, the cells were stained with 0.1% toluidine blue in 1% PFA for 30 min, then rinsed with distilled water several times. Colonies consisting of 50 cells or more were counted as positive and the total number of colonies were expressed per 50 originally seeded cells.

### Differentiation

The SCAP, DPSC, PDLSC and JB-MSC, from all three patients, were differentiated towards the osteogenic- and adipogenic lineages.

#### Adipogenic differentiation

Early passages (P1) of SCAP, DPSC, PDLSC and JB-MSC from all three patients were individually plated in 24-well plates at a density of 1.8 × 10^5^ cells/well. Once the cells were approximately 60% confluent, the adipogenic differentiation protocol was initiated. The protocol was continued for 5 weeks. The growth medium was switched to α-MEM and GlutaMax (GIBCO/Invitrogen, Carlsbad, CA) supplemented with 10% FBS, 1% penicillin–streptomycin solution, 10% FBS, 1 µM dexamethasone, 0.5 mM 3-isobutyl-1-methylxanthine (IBMX), 100 µM indomethacin, and 10 µg/ml insulin (all from Sigma-Aldrich, St Louis, MO). The medium was changed every other day. On days 7, 15, 23 and 31, beginning from the induction, only insulin was added to the medium. At day 35, RNA was extracted from some of the cultures (see below) and the other cultures were stained with Oil Red O. The medium was removed and the cells were washed twice with phosphate buffered saline (PBS) and then fixed with 4% paraformaldehyde (PFA) for 40 min. The cells were then again twice washed with PBS and once with 60% isopropanol before the Oil Red O solution (0.36% oil red O dissolved in 60% isopropanol) was added for 60 min. The cells were then washed once with 60% isopropanol and twice with PBS before being photographed with a Colorview II digital camera.

#### Osteogenic differentiation

Early passages (P1) of SCAP, DPSC, PDLSC and JB-MSC from all 3 patients were individually plated using 96-well, 24-well and 12-well plates at a density of 10^4^ cells/25 cm^2^. When the cultures were fully confluent osteogenic differentiation was induced by use of an osteogenic differentiation medium. In brief SCAP, DPSC, PDLSC and JB-MSC were cultured in osteogenic medium: DMEM + GlutaMAX + 1 g/l d-Glucose + Pyruvate, 10% FBS, 1% penicillin–streptomycin medium supplemented with 0.1 µM dexamethasone, 0.2 mM l-ascorbate and 10 mM glycerol-3-phosphate. The medium was changed every other day for 5 weeks. A similar culture media without the supplements was used as negative control. At day 14 cell culture supernatants were harvested, centrifuged to remove cell debris and analyzed to assess alkaline phosphatase (ALP) activity. ALP was calculated using a colorimetric assay (ab83369, Abcam, Cambridge, UK) according to the manufacturer’s instructions. After harvesting the cell culture supernatant at day 14, the cells were also harvested to assess ALP activity in cell lysates. For each group three samples were used (n = 3). Alizarin Red staining was performed according to the manufacturers protocol after 5 weeks of stimulation. To evaluate the mineralization both qualitatively and quantitatively the OsteoImage assay (Lonza Walkersville Inc. Walkersville, MD) was used after 5 weeks of osteogenic stimulation, as a complement to the Alizarin Red staining. The mineralization was measured qualitatively using fluorescence microscopy and quantitatively by spectrophotometer at 492 nm excitation and 520 nm emission wavelengths.

### qRT-PCR

RNA was collected at baseline, 1, 3 and 5 weeks of stimulation. RNA was isolated from the cells using the RNeasy kit (Qiagen) and the purified RNA was quantified using a Nanodrop 2000c spectrophotometer (ThermoScientific, Sweden). For quantitative RT-PCR cDNA was prepared from RNA using iScript cDNA Synthesis Kit (Bio-Rad Laboratories Inc. Hercules, CA). The cDNA from each type of MSC from each donor was pooled together making a total of 8 groups (unstimulated and stimulated for each MSC cell-type) and either 1 ng or 4 ng/reaction mix was combined with reagents according to the manufacturers protocol (Bio-Rad Laboratories Inc., Hercules, CA). An overview of primers (Sigma) used in this study is provided in Table 1. qRT-PCR was performed using SsoFast™ EvaGreen Supermix (Bio-Rad) in a CFX96 Optical Cycler and analyzed using the CFX96 manager software. According to the manufacturer’s instructions, denaturation/DNA polymerase activation was processed at 95 °C for 30 s followed by PCR 95 °C for15 s, variable annealing temperatures for 30 s (see Table 1) and repeated for 40 cycles. RPL13a was selected as the housekeeping gene and data were calculated as relative expressions according to the ΔΔC (t) principle.

**Table 1:** Primer sequences for qRT-PCR and annealing temperatures

Factor	Forward primer (5′–3′)	Reverse primer (5′–3′)	°C
Osteocalcin	AGCAAAGGTGCAGCCTTTGT	GCGCCTGGGTCTCTTCACT	63.5
Runx2	CCCGTGGCCTTCAAGGT	CGTTACCCGCCATGACAGTA	63.2
Alkaline phosphatase	GGAACTCCTGACCCTTGACC	TCCTGTTCAGCTCGTACTGC	61.1
RPL13a	AAGTACCAGGCAGTGACAG	CCTGTTTCCGTAGCCTCATG	58.3

### Statistical analysis

For statistical analyses in this study, both *t* test (for single comparison) or one-way analysis of variance (ANOVA, for multiple comparison) followed by post hoc Bonferroni test (Prism^®^, Graph-Pad Software Inc) were used to determine statistical differences between experimental groups. Statistical significance was set as *p < 0.05, **p < 0.01, ***p < 0.001.

## Results

**Fig. 2:**
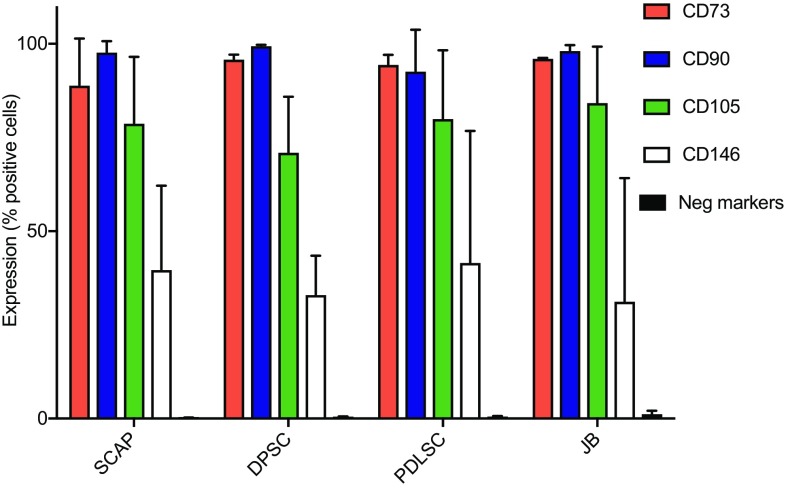
Characterization of MSC by flow cytometry. Flow cytometry analyses of human SCAP, DPSC, PDLSC, and JB-MSC from three different donors at passage 1 are shown (not pooled). All various MSC were positive for expression of CD73, CD90, CD105, CD146 and lacked the expression of the negative markers (HLA-DR, CD45, CD34, CD19 and CD11b). The expression profiles among the tested markers were inter- and intra-individually similar among all various MSCs

Three different patients, undergoing dentoalveolar surgery were selected as donors for the study and MSC from apical papilla, dental pulp, periodontal ligament and jaw bone from each of these donors (illustrated in Fig. 1) were isolated. To ensure that isolated MSC had specific stem cell properties, the cells were initially screened for expression of MSC-specific markers. Flow cytometry analysis for characterization of MSC demonstrated that 75–95% of the cells were positive for expression of CD73, CD90, CD105, and approximately 35–40% of the cells expressed CD146. All MSC, regardless of localization or individual variation, did not express any of negative markers CD11b, CD19, CD34, CD 45 and HLA-DR (expression levels below 0.5% for positive cells). No significant differences were seen between cells from different tissues (Fig. 2).

**Fig. 3:**
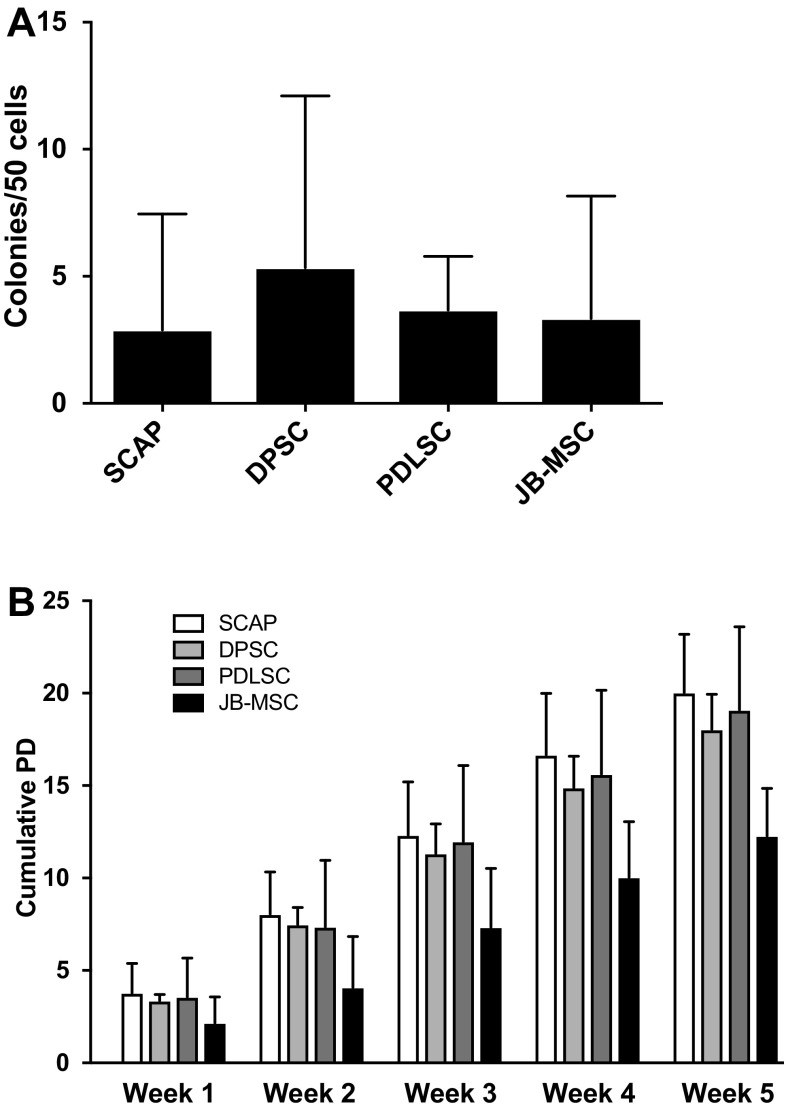
Colony-forming unit-fibroblast (CFU-F) and cumulative population doubling (PD). **A** CFU-F was determined after two weeks of culturing with a starting density of 50 cells per well. Total n = 9 with 3 replicates of each cell type/donor. No significant difference was detected between the groups. **B** Cumulative population doublings were calculated over 5 weeks of culture. No significant differences were detected between the various types of MSC at each week (1–5)

The CFU-F assay showed no significant differences between the four tested groups (SCAP, DPSC, PDLSC, JB-MSC) after 14 days of culturing in α-MEM containing 15% FCS (Fig. 3A). The growth rates of the cells were determined over a 5 week time course. The cumulative population doubling analysis showed no significant difference between the groups. JB-MSC had a tendency of growing slightly slower than the other MSC (Fig. 3B).

**Fig. 4:**
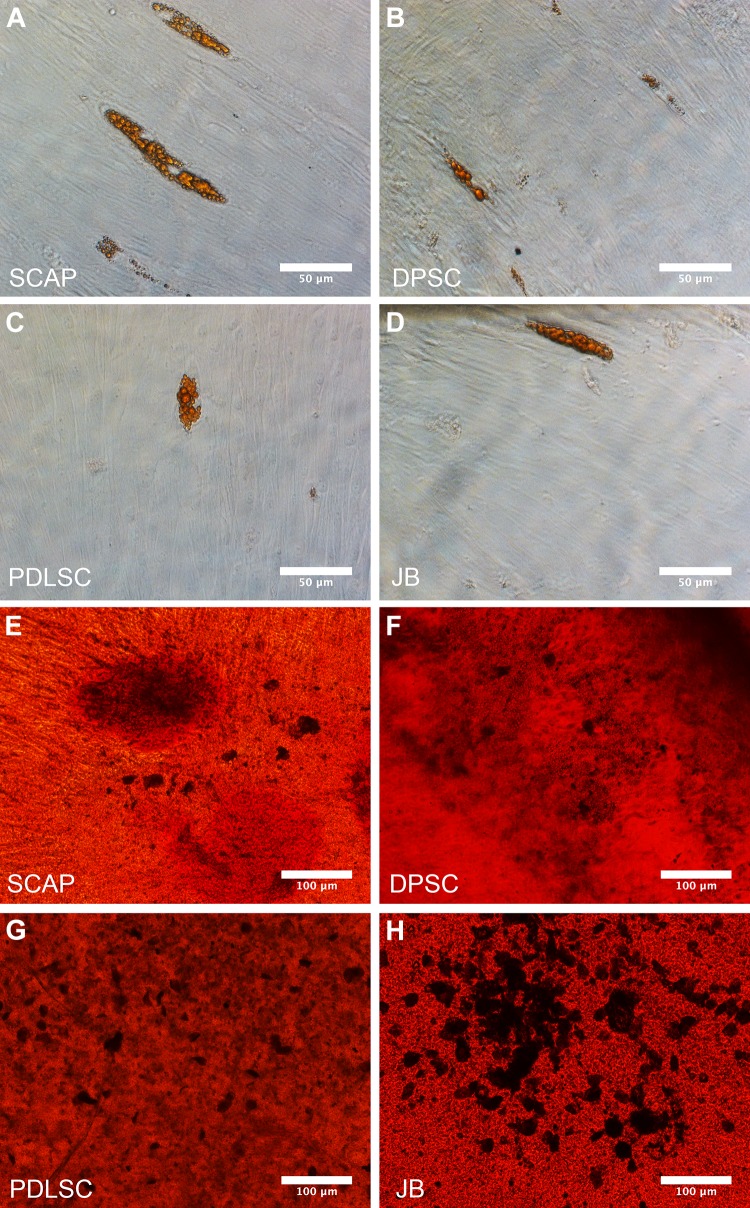
Differentiation of MSC. Oil red O staining of adipogenically differentiated SCAP **A**, PSC **B**, PDLSC **C** and JB-MSC **D**. All cell types were able to differentiate into adipocytes after culturing in adipogenic culture medium after 5 weeks. *Scale bar* 50 μm. A representative figure from each group is shown. Osteogenic differentiation of SCAP **E**, DPSC **F**, PDLSC **G** and JB-MSC **H** shown by Alizarin Red staining. The staining shows the mineralization nodules (*dark spots*) after 5 weeks of culturing in osteogenic medium. *Scale bar* 100 μm. A representative *figure* from each group is shown

We next determined the differentiation abilities of the cells. After 5 weeks of culturing in adipogenic medium all cell types could differentiate into adipocytes, indicated by Oil Red O-uptake (Fig. 4A–D). However, the induced adipogenesis was much more limited compared to what was observed for induced osteogenesis (Fig. 4E–H), illustrated by Alizarin red uptake after 5 weeks of osteogenic stimulation protocol. Cells in all groups formed mineralized nodules after 5 weeks of culturing in the osteogenic medium (Fig. 4E–H). The corresponding control groups cultured in regular medium (unstimulated) did not show any signs of adipogenic nor osteogenic differentiation after 5 weeks (data not shown).

**Fig. 5:**
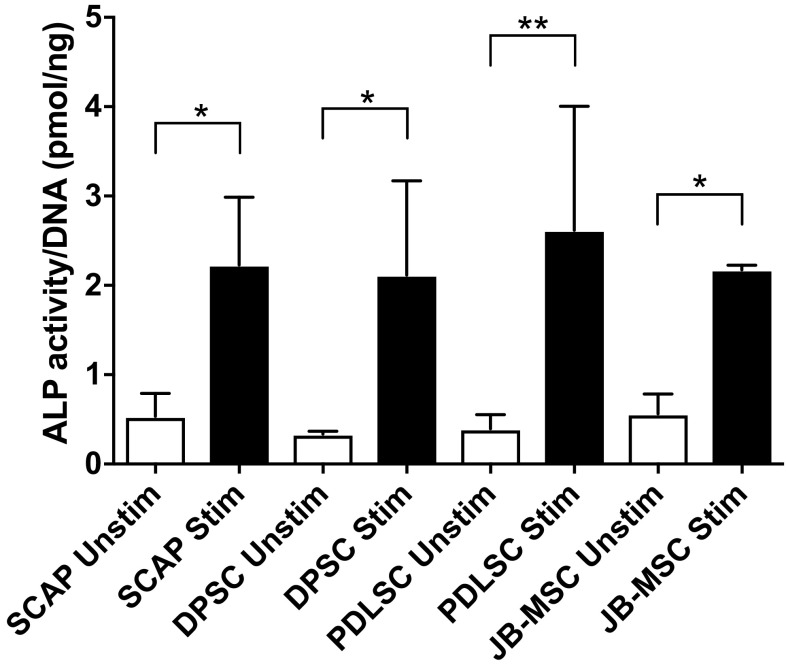
Alkaline phosphatase (ALP) activity. ALP activity was assessed after two weeks of *in vitro* culturing in osteogenic medium. There was a significant difference in ALP activity between the stimulated groups and the unstimulated but no significant difference between the various MSC groups. All data are normalized to the amount of DNA. *Asterisk* show the level of significance compared to each corresponding control (i.e. unstim vs stim); *p < 0.05, **p < 0.01. For each group three samples were used (n = 3). Mean values ±SD are indicated. No significant differences were seen in the ALP expression between the stimulated groups

Since our interest was focused on osteogenic differentiation of the various cell types we explored this in more detail. Firstly, we used an alkaline phosphatase (ALP) assay. Supernatants and cell lysates were harvested after 14 days of osteogenic stimulation. No ALP activity was observed in the supernatants from the unstimulated or osteogenic stimulated cells (data not shown). In contrast, in the cell lysates all the stimulated groups showed significantly elevated ALP activity two weeks after induction, compared with unstimulated cell lysates. No significant differences were detected between stimulated groups (Fig. 5).

**Fig. 6:**
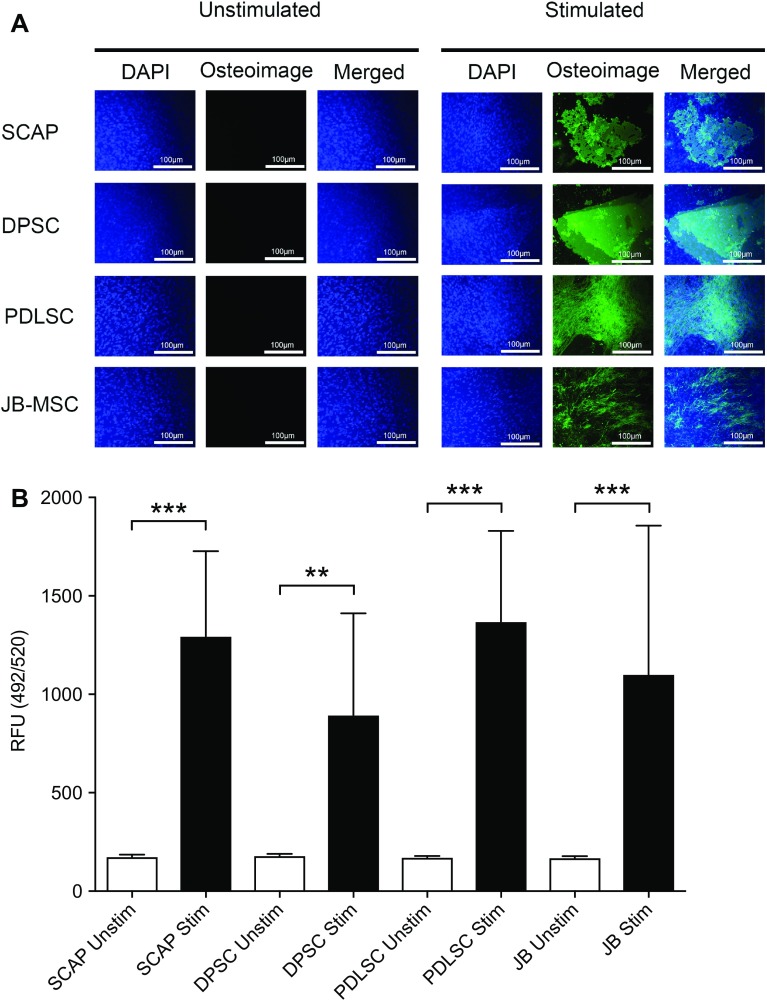
Qualitative and quantitative assessment of osteogenic differentiated MSC. *In vitro* differentiation of SCAP, DPSC, PDLSC and JB-MSC into osteoblasts determined by OsteoImage™ assay after 5 weeks of induction. **A** Fluorescent microcopy (×10 magnification) of the cells revealed the fluorescent staining reagent in the assay could only bind to hydroxyapatite of the bone nodules in the stimulated groups. The unstimulated cells did not show any mineralization. DAPI (4′,6-diamidino-2-phenylindole) was used to counter-stain the fixed cells. A representative image from each group is shown. *Scale bar* 100 µm. **B** The fluorescence staining in the assay is proportional to the amount of mineralization and quantitative assessment could be made from a parallel experiment in 96 well plates with no DAPI staining. By use of a spectrophotometer (492 nm excitation and 520 nm emission) the amount of fluorescent staining binding to the hydroxyapatite produced by the osteostimulated cells was measured and calculated. The amount is expressed in relative fluorescent units (RFU), showing a significantly higher hydroxyapatite content in the stimulated groups compared with the unstimulated. *Asterisk* show the level of significance compared to each corresponding control (i.e. unstim vs stim); **p < 0.01, and ***p < 0.001, n = 9 for each group (originating from 3 donors per group). Mean values ±SD are indicated. Multiple comparisons between the stimulated groups showed no significant difference in hydroxyapatite content between any of the groups

To further analyze the osteogenic capacity of the collected MSC, we utilized a qualitative and quantitative assay (Osteoimage™). The formation of mineralized nodules deposited by the osteostimulated cells was visualized by the binding of a fluorescent staining reagent to the hydroxyapatite portion in the Osteoimage assay. The mineralization was assessed qualitatively by fluorescence microscopy (Fig. 6A). The unstimulated cells did not take up any fluorescent staining dye in any tested group, while all stimulated cells significantly increased their uptake of the green fluorescent dye (Fig. 6A). The amount of green fluorescent staining is proportional to the amount of mineralization in the cell culture and this was quantified by spectrophotometry (Fig. 6B). Significant differences were detected between each unstimulated and stimulated group. In contrast, no statistical differences were observed when the stimulated groups were compared with each other (Fig. 6B).

**Fig. 7:**
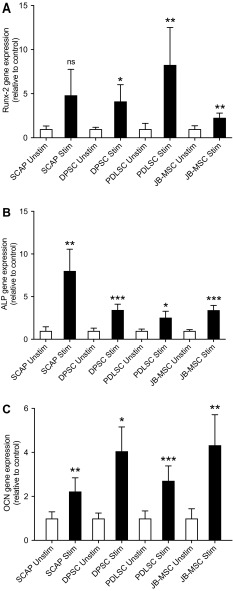
Osteogenic gene expression of various MSC. The relative expression of osteogenic gene markers Runx2, ALP and OCN was assessed by qRT-PCR for unstimulated (unstim) and stimulated (stim) SCAP, DPSC, PDLSC, and JB-MSC. **A** Expression of **Runx-2** after 1 week of osteogenic stimulation. **B** Expression of **ALP** after 3 weeks of osteogenic induction. **C** Expression of **OCN** after 5 weeks of osteogenic stimulation. Relative expression levels are shown with regard to control unstimulated samples (value = 1) and normalized against the reference gene RPL13a. *Asterisk* show the level of significance compared to each corresponding control (i.e. unstim vs stim); *p < 0.05, **p < 0.01, and ***p < 0.001, n.s., not significantly different. n = 3–6 from pooled RNA samples (originating from 3 donors per group). Mean values ± SD are indicated. Multiple comparisons between the stimulated groups (SCAP, DPSC, PDLSC, and JB-MSC) showed no significant differences for the expression of Runx2 except for PDLSC vs. JB-MSC, where a weak significance was seen (*p* = 0.02). Regarding ALP expression in the stimulated groups, SCAP showed a significantly higher expression (*p* < 0.001) than all the other stimulated groups. There were no significant differences in expression between the other groups. The expression of OCN was significantly higher for stimulated PDLSC vs. stimulated JB-MSC (*p* = 0.009), but no significances in expression of OCN were seen between any of the other stimulated groups

We also investigated gene changes during the stimulation with osteogenic media. The relative expression of Runx-related transcription factor 2 (Runx-2) was increased in all four groups after 1 week of culturing in osteogenic medium (Fig. 7A). After 3 weeks of osteogenic stimulation there was a significant elevation of the gene expression levels of ALP in all stimulated groups compared with their corresponding unstimulated control cells (Fig. 7B). After 5 weeks of culturing in osteogenic medium the relative expression of Osteocalcin (OCN) was increased in all groups with a statistical difference between the stimulated and unstimulated cells but no statistically significant difference between the different stimulated groups (Fig. 7C).

## Discussion

MSC are a heterogeneous group of immature cells that can be isolated from various tissues and are able to differentiate into different types of specialized cells depending on the stimuli and culture conditions under which they are expanded. They also show a low immunogenicity and an immunosuppressive activity [31, 32]. All these features make MSC highly interesting for cell therapy applications. MSC isolated from different tissues show a lot of similarities, such as the capability for self-renewal, clonogenic capacity and multipotent differentiation but they also have differences. For example, MSC from bone marrow can more easily be differentiated into osteoblasts than MSC isolated from fat tissue [15]. MSC isolated from bone marrow also have differences depending on harvest site suggesting there is a site dependent factor based on different embryological origins. MSC from jawbone have a higher proliferation rate than BM-MSC harvested from iliac crest and seem to have a better self-renewal capacity [16]. On the contrary, BM-MSC from iliac crest can more easily undergo adipogenic differentiation compared to MSC from jawbone [16]. These observations indicate that the jaws can be a good source of MSC for future bone regeneration therapies. Other previous research has indicated the benefits of MSC from dental tissues such as dental pulp [22], periodontal ligament [21] and apical papilla [25, 26]. Thus in the present study, we aimed to compare MSC from jawbone with other MSC from the oral cavity.

All aforementioned MSC share the same developmental origin, but individual differences among MSC-donors are well known. We isolated various MSC from the same donors in order to minimize the risk of individual variation between the respective MSC. We collected the dental-MSC; SCAP, DPSC and PDLSC according to previously described isolation methods [29]. In addition, in this study we also isolated MSC from jawbone by utilizing the surgically removed cortical bone in order to get access to the tooth. We did not harvest any trabecular bone due to the higher risk of morbidity such as nerve injury. The collected bone was limited in size (approximately 3 mm^3^) and consisted of dense cortical bone in all three donors. We did not focus on comparing if there could be any age or gender specific differences among the three MSC-donors that would affect their osteogenic capacity.

In this study we relied on the plastic adherence capacity for isolation of the MSCs. There are methods to further improve the isolation procedure by using imunomagnetic selection based on the expression of MSC associated surface markers STRO-1 antigens to get a more homogenous population of MSC [33]. The characterization by flow cytometry showed the same expression patterns for the tested positive markers (CD73, CD90, CD105 and CD146) and lack of expression for negative markers (CD11b, CD19, CD34, CD 45 and HLA-DR) for JB-MSC compared with SCAP, DPSC and PDLSC. All cell types were able to undergo adipogenic- and osteogenic differentiation depending on culture conditions. However, the main focus of this study was to investigate the osteogenic capacity and investigate any differences among the isolated MSC.

Alizarin Red staining that is often used for osteogenic evaluation, detects calcium accumulation and formation of chelates. However, the differentiation into osteoblasts is marked by the formation of mineralized inorganic hydroxyapatite nodules (Ca_10_(PO_4_)_6_(OH)_2_). To be able to evaluate the mineralization both qualitatively and quantitatively we utilized another assay as complement to the Alizarin Red staining. The Osteoimage™ assay, used in this study, contains a staining agent that binds to the hydroxyapatite portion of the mineralized nodules and thereby can truly reflect the levels of mineralized tissue. We did not find any significant differences in osteogenic capacity of JB-MSC compared with SCAP, DPSC, or PDLSC.

Elevated ALP levels were seen after 14 days in cell lysates of all stimulated groups with no significant differences between the different cell types. JB-MSC showed a similar capacity for ALP activity as seen in the dental MSCs (SCAP, DPSC, PDLSC). There were significant differences between the unstimulated and stimulated cells.

The induced osteogenic differentiation of the MSC was further measured by qRT-PCR to assess the relative expression of Runx2, ALP and OCN. ALP is a commonly used marker for osteogenic activity. ALP is expressed early in the differentiation of osteoblasts and later in the development it declines, while other genes, such as OCN, are upregulated [34]. The most important regulatory pathways in control of osteoblastic differentiation and ALP expression are the BMP/RUNX2/Osterix system and the WNT signaling cascade [35]. Runx2 is vital for the commitment of MSC to the osteoblast lineage and of great influence in the early stages of osteoblast differentiation but has to be down-regulated for further bone maturation [36]. Hence, we selected different time points to examine the various gene expressions and their changes due to the stimulation protocol. After one week of osteogenic stimulation we observed an upregulation of Runx2 in all groups indicating an early stage of osteogenic differentiation. Regarding ALP gene expression, significant upregulation was detected in all stimulated MSC compared with the unstimulated ones. Comparing the stimulated groups we did observe a significantly higher expression for ALP in SCAP compared to all the other groups. No significant differences were seen between the other stimulated groups. This could suggest a faster differentiation for SCAP compared to the other, possibly more mature groups. Finally, to be sure of the effects seen after five weeks we measured the ultimate marker (OCN) for osteogenic differentiation. After five weeks, the expression of OCN was also elevated significantly in all stimulated groups. No significant difference was seen between the various stimulated dental MSC, however PDLSC showed a significantly higher expression of OCN compared to JB after 5 weeks of osteogenic stimulation. Collectively, our conclusion is that JB-MSC are as good as SCAP, DPSC, and PDLSC in their capacity of osteogenic differentiation *in vitro*. However it seems that they are growing a bit slower.

This conclusion is based on the results from the proliferation study together with ALP test, Osteogenic assay (OsteoImage) and qRT-PCR.

So, what is the rationale of selecting JB-MSC? Compared with SCAP, the harvest of JB-MSC is not age dependent, since SCAP can only be isolated from a tooth with developing root. To collect the SCAP it is necessary to extract the developing tooth, explaining why most of the previous studies on SCAP refer to surgically removed retained teeth. To collect DPSC, it is necessary to devitalize a tooth or extract it as well, and the collection of SCAP and PDLSC requires the tooth to be extracted or there is a major risk of damage to the root surface leading to future resorption of the root. With these clinical limitations in mind, it can be concluded that the harvesting of all of the dental-MSC are more invasive than the isolation of JB-MSC. Furthermore, as we have shown in this study only a small volume of the jawbone is sufficient for isolation of JB-MSC. These cells can then be expanded *in vitro* and their osteogenic potential appears to be similar to dental-MSCs. Another rationale of using JB-MSC for craniofacial bone regeneration applications, is the possibility that the site-specific properties would then be retained and unwanted properties could be avoided.

There are several studies comparing the osteogenic differentiation capacity of different dental MSC [30, 37, 38]. Several studies have also tried to compare various dental-MSC with BM-MSC and ASC [15, 29, 39]. There are also studies comparing BM-MSC from appendicular bones with MSC from jawbone [16]. To our best knowledge, the present study is the first of its kind to compare JB-MSC with various dental MSC that are collected from the same individuals. We clearly show that JB-MSC have at least similar osteogenic capacity as dental-MSC *in vitro*. Clinically, free non-vascularized cortical bone transplants are already being harvested from intraoral donor sites [5, 6, 40]. JB-MSC is easy to harvest in a minor surgical procedure with a low risk of morbidity, making it a good alternative to dental-MSC in bone regeneration therapies, in particular for jaw-bone reconstructions. Future *in vivo* experiments will be needed to fully compare the osteogenic differentiation capacity of the various MSCs.
